# Multiparasitism enables a specialist endoparasitoid to complete parasitism in an unsuitable host caterpillar

**DOI:** 10.1038/s41598-025-91403-3

**Published:** 2025-03-11

**Authors:** Kazumu Kuramitsu, Yooichi Kainoh, Kotaro Konno

**Affiliations:** 1 Faculty of Life and Environmental Sciences, University of Tsukuba, Tennodai 1-1-1, Tsukuba, Ibaraki 305-8572 Japan; 2 Institute of Agrobiological Sciences, National Agriculture and Food Research Organization (NARO), 1‑2 Owashi, Tsukuba, Ibaraki 305‑8634 Japan; 3 Japan Society for the Promotion of Science, 5-3-1 Kojimachi, Chiyoda-ku, Tokyo, 102-0083 Japan

**Keywords:** Kleptoparasitism, Pirate parasitism, *Cotesia kariyai*, *Meteorus pulchricornis*, Multiparasitism, *Mythimna loreyi*, Behavioural ecology, Animal behaviour, Entomology

## Abstract

Parasitoid wasps serve as natural enemies of numerous insect species; therefore, knowledge of host-parasitoid interactions is fundamental for understanding ecosystems. Each endoparasitoid wasp taxon exhibits a specific host range. Female parasitoids, however, occasionally oviposit into non-host species. Since the survival probability of eggs in non-host species is virtually zero, these behaviors have long been considered maladaptive. However, in the present study, we found that eggs of a specialist parasitoid, *Cotesia kariyai* (Hymenoptera: Braconidae), oviposited in unsuitable host caterpillars, *Mythimna loreyi* (Lepidoptera: Noctuidae), successfully complete larval development in the non-host when these caterpillars are simultaneously oviposited by another naturally sympatric parasitoid wasp, *Meteorus pulchricornis* (Hymenoptera: Braconidae), for which *My*. *loreyi* is the usual host. This observation suggests that the seemingly maladaptive behavior of ovipositing in unsuitable host insects can be adaptive, allowing them to maintain reproductive potential in environments where their ordinary hosts are absent. We propose a new term, “pirate parasitism”, for this type of obligatory multiparasitism. Understanding detailed mechanisms of this phenomenon may provide deeper insights into parasitoid-host dynamics and evolution of host use strategies by parasitoids.

**Supplementary Information:**

The online version contains supplementary material available at 10.1038/s41598-025-91403-3.

## Introduction

Parasitoid wasps comprise one of the most species-rich animal groups. They serve as natural enemies of numerous insect species, and are widely recognized as important in both natural and agricultural ecosystems^1–3^. Each parasitoid species exhibits a more or less specific host range; thus, in the context of ecological, evolutionary, and agricultural science, host-parasitoid associations are essential to understand biological networks in natural and agricultural ecosystems^1,2,4–7^.

Parasitoids, which deposit their eggs in bodies of host insects and develop inside them, known as endoparasitoids, must overcome host immunity to complete their development^1^. Endoparasitoid wasps employ various maternal strategies to protect their eggs and larvae from host immune systems. For instance, females of ichneumonoid endoparasitoids (Hymenoptera: Braconidae and Ichneumonidae) utilize venom, a mixture of proteinaceous and non-proteinaceous components, and/or polydnaviruses, symbiotic, large, double-stranded DNA viruses, to suppress host immunity^8–13^. Anti-host venoms and viruses exhibit specificity depending on host species or strain; therefore, such specificity restricts host ranges of endoparasitoids^14–16^. In fact, parasitoid eggs are encapsulated and are usually killed by host cells when deposited in non-host species^17–19^. Consequently, female parasitoids may have evolved host-location and host-acceptance strategies using physical and chemical cues to oviposit in suitable host species^20–23^.

However, female parasitoids encounter numerous sympatric non-host species (species unsuitable for the parasitoid to complete development). Occasionally, parasitoid females are attracted to cues derived from non-host species, either chemically or physically^24–29^, so that they accept the non-host species, and oviposit onto or into them^24,30–35^. Since the survival probability of parasitoid eggs in non-host species has been regarded as essentially zero, these behaviors have been considered as “misunderstandings of information”^28^, “accidents”^2^ or “mistakes”^36^ by parasitoid females. However, in the context of parasitoid host range evolution, theory predicts that these “mistakes” may lead to host shifts or host range expansion at the population level when parasitoids fortuitously attack potential new host species that are suitable for parasitoid development. This occurs through associative learning by newly emerged female parasitoids, resulting in a preference for oviposition in the same species in which they developed^2,37–39^. However, despite the potentially significant role of such “mistakes” in evolution of parasitoid host range, their adaptive significance has been little discussed.

Kraaijeveld^31^ hypothesized that oviposition by female parasitoids into non-host species could potentially increase the fitness of parasitoids in the absence of their ordinary host species. The scenario proposed was as follows. Eggs of the parasitoid oviposited in a non-host species might be able to complete their development if the non-host insects had previously been parasitized by a sympatric parasitoid species that uses the same insect species as its ordinary host. In other words, the endoparasitoid might engage in kleptoparasitism by exploiting anti-host immunity conferred by another parasitoid species via multiparasitism in unsuitable host species. To test this hypothesis, Kraaijeveld^31^ used larvae of *Drosophila simulans* (Diptera: Drosophilidae), an endoparasitoid, *Asobara tabida* (Hymenoptera: Braconidae), and another endoparasitoid, *Leptopilina boulardi* (Hymenoptera: Figitidae). The study revealed that even if *A*. *tabida* accepted *D*. *simulans* larvae and oviposited into them, its offspring could not complete development in those larvae. However, when the host larva had previously been oviposited by *L*. *boulardi*, approximately 15% of *Asobara* larvae survived in *D*. *simulans* larvae for 4 or 6 days after oviposition. Based on these results, the author concluded that the response and oviposition of female parasitoids into non-host species could indeed benefit parasitoids because it could result in reproductive success in the context of multiparasitism^31,40^. Similarly, Vinson^41^ also conducted multiparasitism experiments using the parasitoid, *Cardiochiles nigriceps* (Hymenoptera: Braconidae), its non-host caterpillar, *Heliothis zea* (Lepidoptera: Noctuidae), and a parasitoid *Microplitis croceipes* (Hymenoptera: Braconidae), which uses *H*. *zea* as its ordinary host. The author demonstrated that parasitism by *M*. *croceipes* inhibits the immune response of *H*. *zea* to *C. nigriceps* eggs/larvae.

As far as we know, successful parasitism in unsuitable hosts through multiparasitism with other parasitoids has been reported in several insect systems^32,42–44^, such as *Cotesia flavipes* (Hymenoptera: Braconidae)^32^ and *Psyttalia fletcheri* (Hymenoptera: Braconidae)^44^. In all of these cases, parasitoids showed successful parasitism in non-host species only when the host had been previously oviposited by another parasitoid species. However, all the previous examples involved interactions between parasitoid species, at least one of which was introduced into the host range^32,42,43^. Even if the ecological significance of such systems may apply in the context of biological invasions, Kraaijeveld’s hypothesis has never been examined in herbivore-parasitoid systems that are naturally sympatric and share a long evolutionary history. To test Kraaijeveld’s hypothesis, successful parasitism in non-host species via multiparasitism should be examined in herbivore-parasitoid systems that satisfy the following conditions: (1) a potential host species and two parasitoid species are naturally sympatric and occur simultaneously in the field, (2) both parasitoids have the capacity to search for and oviposit into the potential host species, (3) the potential host species is unsuitable for one of the parasitoids due to its immune response when parasitized by that species alone, while being a suitable host for the other parasitoid.

Here, we tested Kraaijeveld’s hypothesis using the following naturally sympatric insects in laboratory experiments: two endoparasitoids, *Cotesia kariyai* (hereafter, *Ck*) (Hymenoptera: Braconidae) and *Meteorus pulchricornis* (hereafter, *Mp*) (Hymenoptera: Braconidae), and larvae of a moth, *Mythimna loreyi* (hereafter, *Myl*) (Lepidoptera: Noctuidae).

*Ck* is a monophagous specialist, and a gregarious endoparasitoid of *Mythimna separata* (hereafter, *Mys*) (Lepidoptera: Noctuidae) caterpillars^45^. However, *Ck* females respond to cues related to sympatric unsuitable host lepidopteran larvae, including *Myl*, and oviposit into them, but no offspring successfully develop and emerge^24,28,34,46^. The other parasitoid, *Mp*, is a generalist, solitary larval endoparasitoid of numerous species of exophytic lepidopteran caterpillars, including *Myl*^47,48^.

These two parasitoids are comprehensively studied for biological characteristics, such as general bionomics, host searching behavior, oviposition behavior, and immune suppression mechanisms^46,48–55^. In addition, multiparasitism of *Ck* and *Mp* in *Mys*, a suitable host for both parasitoids, is also well documented^56–58^.

Caterpillars of both *Myl* (an unsuitable host for *Ck*, but a suitable host for *Mp*) and *Mys* (a suitable host for both parasitoids) are external leaf feeders that feed primarily on grasses. *Myl* and *Mys* are frequently sympatric and frequently co-occur in maize or sorghum fields in South, Southeast, and East Asian countries^59–62^. They are morphologically, behaviorally, and ecologically very similar; in terms of body size, color pattern, range of host plants, and diurnal larval behavior. However, they can be distinguished by morphology of adults and hairs on the larval head capsule^59,62–64^. To the best of our knowledge, there are no records of *Ck* emerging from *Myl*. However, reports of parasitoids from *Myl* in Japan are extremely scarce, and as mentioned earlier, the external morphologies of *Myl* and *Mys* are very similar. Consequently, there is a possibility that records of *Ck* emerging from *Myl* may have been mistakenly attributed to *Mys*.

In this study, we tested Kraaijeveld’s hypothesis by addressing the following questions: (i) Do females of both parasitoid species accept unparasitized *Myl* caterpillars or *Myl* caterpillars previously oviposited by the heterospecific parasitoid species as targets for oviposition? (ii) Do *Ck* eggs oviposited in *Myl* caterpillars show successful parasitism, when multiparasitism with *Mp* occurs? (iii) Does the order and interval of oviposition by the two parasitoid species affect the outcome of multiparasitism? (iv) Can successful parasitism in non-hosts occur through multiparasitism in simultaneous free-oviposition tests in cages? Additionally, larvae of both parasitoids in multiparasitized *Myl* caterpillars were observed by dissection to ensure that hyperparasitism by both parasitoid species (cf.^65^) did not occur.

Here, we report successful parasitism by a specialist parasitoid in an unsuitable host species via multiparasitism in a naturally sympatric insect system. Based on our results, we discuss the ecological significance of erroneously responding and ovipositing in unsuitable host species. We propose a new term, “pirate parasitism”, for this type of parasitism.

## Results

### Experiment 1: host acceptance and oviposition

Regardless of the host instar or previous oviposition by the heterogeneous parasitoid, all *Ck* females exhibited oviposition behavior towards *Myl* caterpillars, similar to their behavior toward their usual host, *Mys* (Supplementary Figure S1a). The number of *Ck* eggs in oviposited *Myl* caterpillars, ranged from 53.7 ± 17.8 to 59.0 ± 18.3 (mean ± SD, *n* = 20 for each), and did not vary significantly among host instars or in relation to previous parasitism. The number of *Ck* eggs deposited did not differ significantly from those in the usual host, *Mys* (Supplementary Figure S1b, ANOVA, *F*_6, 133_ = 0.19, *p* > 0.05). Similarly, over 85.0% (*n* = 30 for each) of *Mp* females accepted *Myl* caterpillars for oviposition, regardless of the host instar or previous oviposition by the heterogeneous parasitoid, and the difference in the host acceptance rate did not differ significantly among *Myl* instars or in relation previous parasitism (χ^2^ = 3.41, df = 5, *p* > 0.05, Supplementary Figure S1c). A single *Mp* egg was consistently oviposited in *Myl* caterpillars without exception (Supplementary Figure S1d).

### Experiment 2: single-parasitism experiments

Regardless of the host instar, *Ck* never emerged from *Ck*-oviposited *Myl* caterpillars (Supplementary Figure S2a, *n* = 200 for each). Among *Ck*-oviposited *Myl* caterpillars, 83.0–86.5% of larvae survived until pupation, whereas the remainder died without emergence of *Ck* (Supplementary Figure S2a). In contrast, *Mp* successfully emerged from 76.7 to 86.7% of *Mp*-oviposited *Myl* caterpillars (Supplementary Figure S2b, *n* = 30 for each). The difference in the *Mp* emergence rate did not differ significantly among *Myl* instars (χ^2^ = 1.25, df = 2, *p* > 0.05, Supplementary Figure S2b).

### Experiment 3: multiparasitism experiments in different host instars

**Fig. 1:**
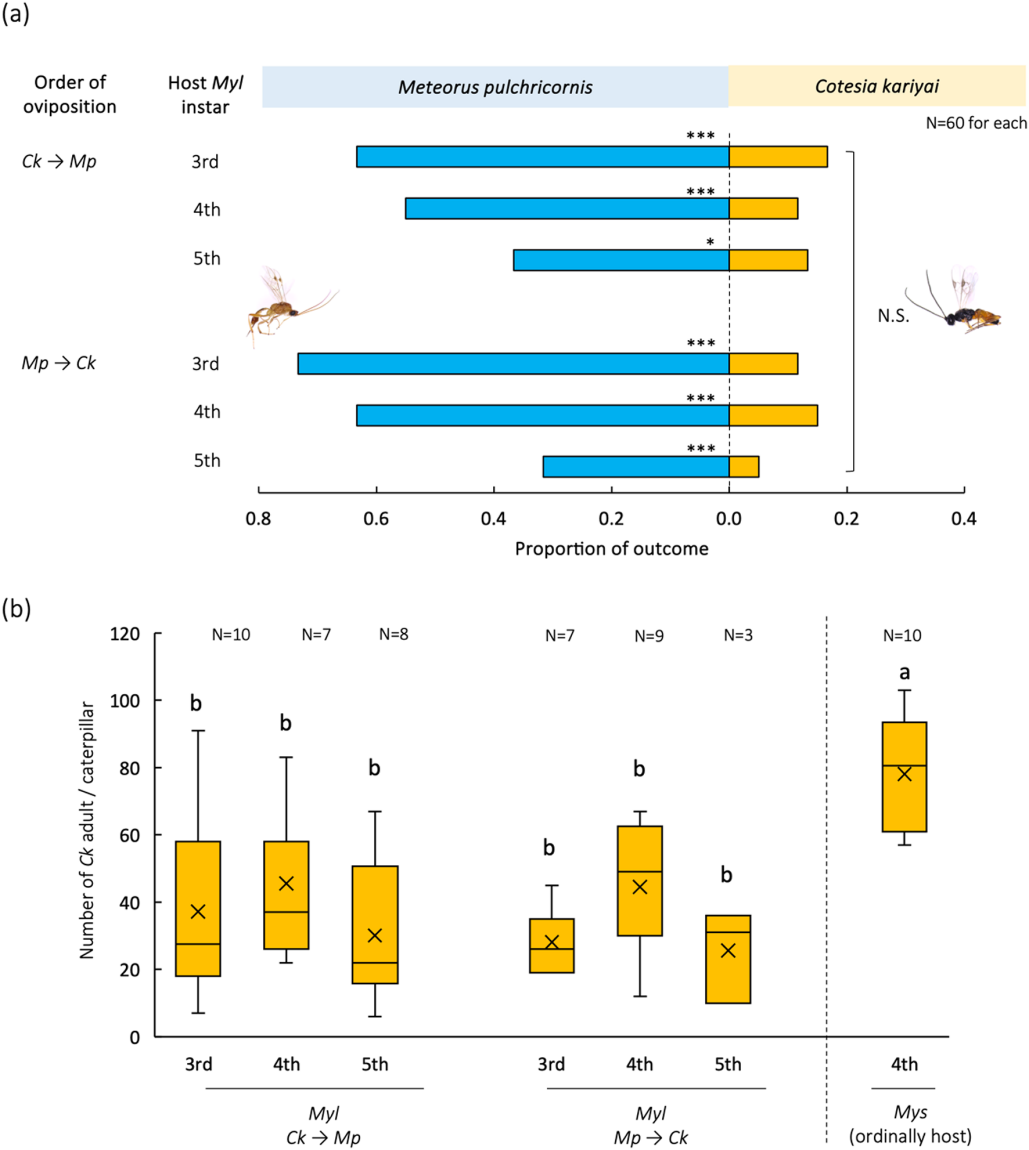
Outcome of multiparasitism by *Cotesia kariyai* (*Ck*) and *Meteorus pulchricornis* (*Mp*) in *Mythimna loreyi* (*Myl*) caterpillars. The successful parasitism rate of each parasitoid in 3rd, 4th or 5th instar *Myl* caterpillars (**a**), and number of emerged wasps per caterpillar when *Ck* emerged from the caterpillars (**b**). *Mys* = *My*. *separata*. (∗*P* < 0.05; ∗∗*P* < 0.01 and ∗∗∗*P* < 0.001 by binomial test; N.S.: no significant differences by Chi-square test, Bars labeled with the same letter are not significantly different on the basis of Tukey’s HSD test after ANOVA)

Among multiparasitized caterpillars, parasitoid wasps emerged from 50.0 to 90.0% of the caterpillars (Fig. 1a). Regardless of host instar or order of oviposition, no instances were observed in which both parasitoid wasps emerged from a single larva. Interestingly, *Ck* emerged from 5.0 to 15.0% of *Myl* caterpillars (Fig. 1a). The successful parasitism rate of *Ck* did not differ among treatments (χ^2^ = 4.56, df = 5, *p* > 0.05). The number of emerged *Ck* adult per *Myl* caterpillar, ranging from 25.7 ± 13.8 to 45.6 ± 21.4 (mean ± SD) on average, was not significantly different among caterpillar instars or order of oviposition. However, significantly fewer *Ck* adults emerged from *Myl* caterpillars than from their usual host, *Mys* (Fig. 1b, ANOVA, *F*_6, 47_ = 7.028, *p* < 0.05, Tukey-Kramer, *p* < 0.05).

Regardless of the host instar or order of oviposition, the successful parasitism rate of *Mp* (31.7–73.3%) was significantly higher than that of *Ck* (Fig. 1a, binomial test, *p* < 0.05). For *Ck* emerging from *Myl*, all 30 *Ck* females (5 per treatment) showed oviposition behavior toward *Mys* caterpillars, and their offspring emerged from the caterpillars. The number of *Ck* adults that emerged from those caterpillars did not differ significantly among treatments (Supplementary Figure S3, ANOVA, *F*_5, 24_ = 0.256, *p* < 0.933).

### Experiment 4: multiparasitism in different sequences and intervals

**Fig. 2:**
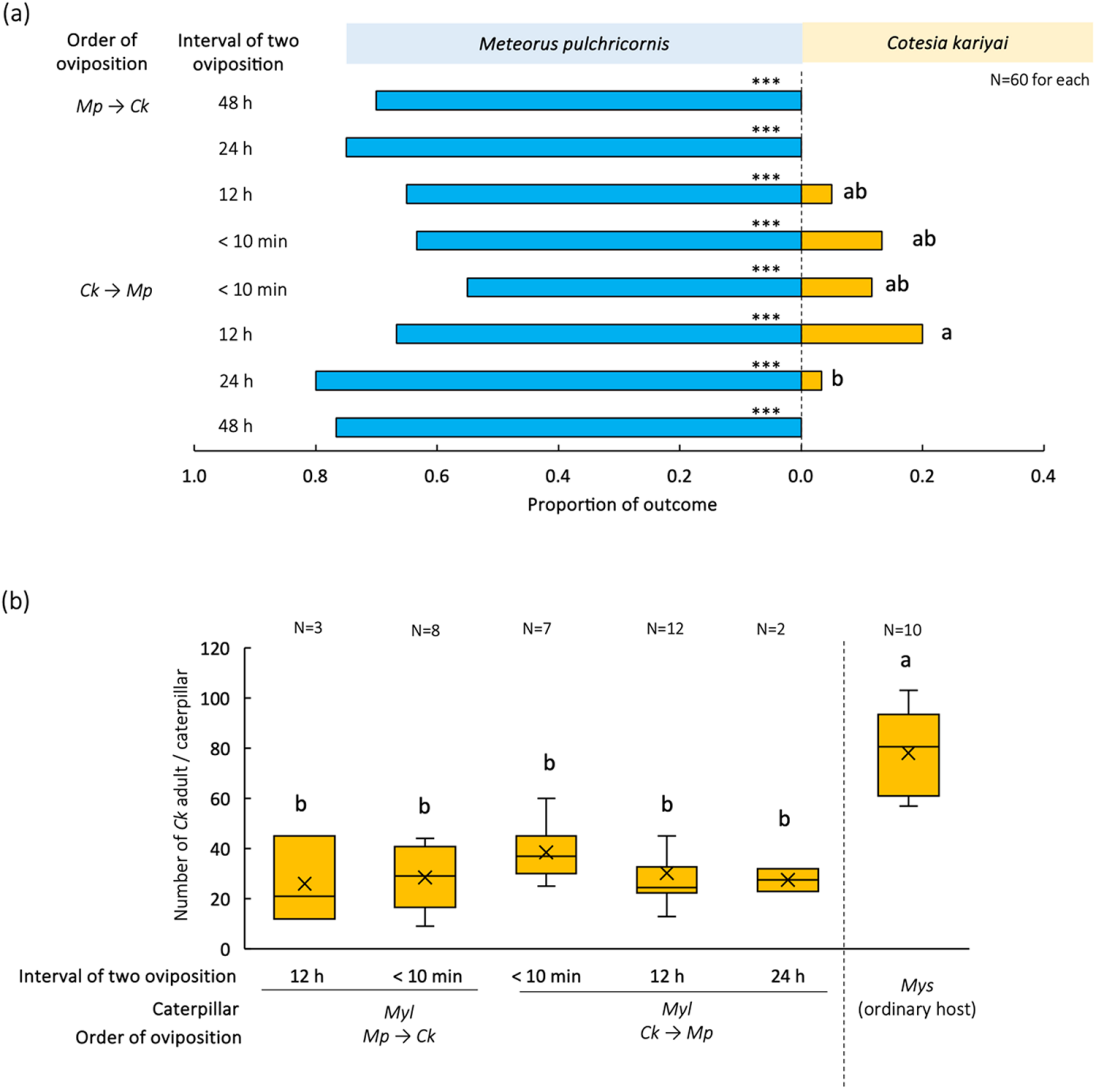
Effects of order and interval of oviposition by two parasitoids on outcomes of multiparasitism. The successful parasitism rate of each parasitoid in 4th instar *Myl* caterpillars (**a**), and numbers of wasps per caterpillar when *Ck* emerged from the caterpillars in different orders and intervals of oviposition by two parasitoids (**b**). *Myl* = *Mythimna loreyi*, *Mys* = *My*. *separata*,* Ck* = *Cotesia kariyai*, and *Mp* = *Meteorus pulchricornis.* (∗*P* < 0.05; ∗∗*P* < 0.01 and ∗∗∗*P* < 0.001 by binomial test; bars labeled with the same letter are not significantly different on the basis of (a) Tukey’s WSD teat after Chi-square test and (b) Tukey’s HSD test after ANOVA.)

Successful parasitism by *Ck* occurred when *Ck* oviposited from 24 h before until 12 h after *Mp* (Fig. 2a). Beyond these times, no successful parasitism by *Ck* was observed. Under experimental conditions in which successful parasitism by *Ck* was observed, the success rate of *Ck* ranged from 5.0 to 20.0%. The latter occurred when *Ck* oviposited 12 h before *Mp*, but the success rate was significantly lower when *Ck* oviposited 24 h before *Mp* (Fig. 2a, χ^2^ = 38.04, df = 7, *p* < 0.05; Tukey’s WSD, *p* < 0.05). The number of emerged *Ck* adults per *Myl* caterpillar, ranging from 26.0 ± 17.1 to 38.4 ± 11.7 (mean ± SD) on average, was not significantly different among conditions, but they were significantly fewer than the number of *Ck* adults that emerged from their usual host, *Mys* (78.1 ± 16.7) (Fig. 2b, ANOVA, *F*_5, 36_ = 16.05, *p* < 0.05, Tukey-Kramer, *p* < 0.05). In all conditions, the successful parasitism rate of *Mp* was significantly higher than that of *Ck* (Fig. 2a, binomial test, *p* < 0.001).

### Experiment 5: observations of parasitoid larvae in multiparasitized caterpillars

**Fig. 3:**
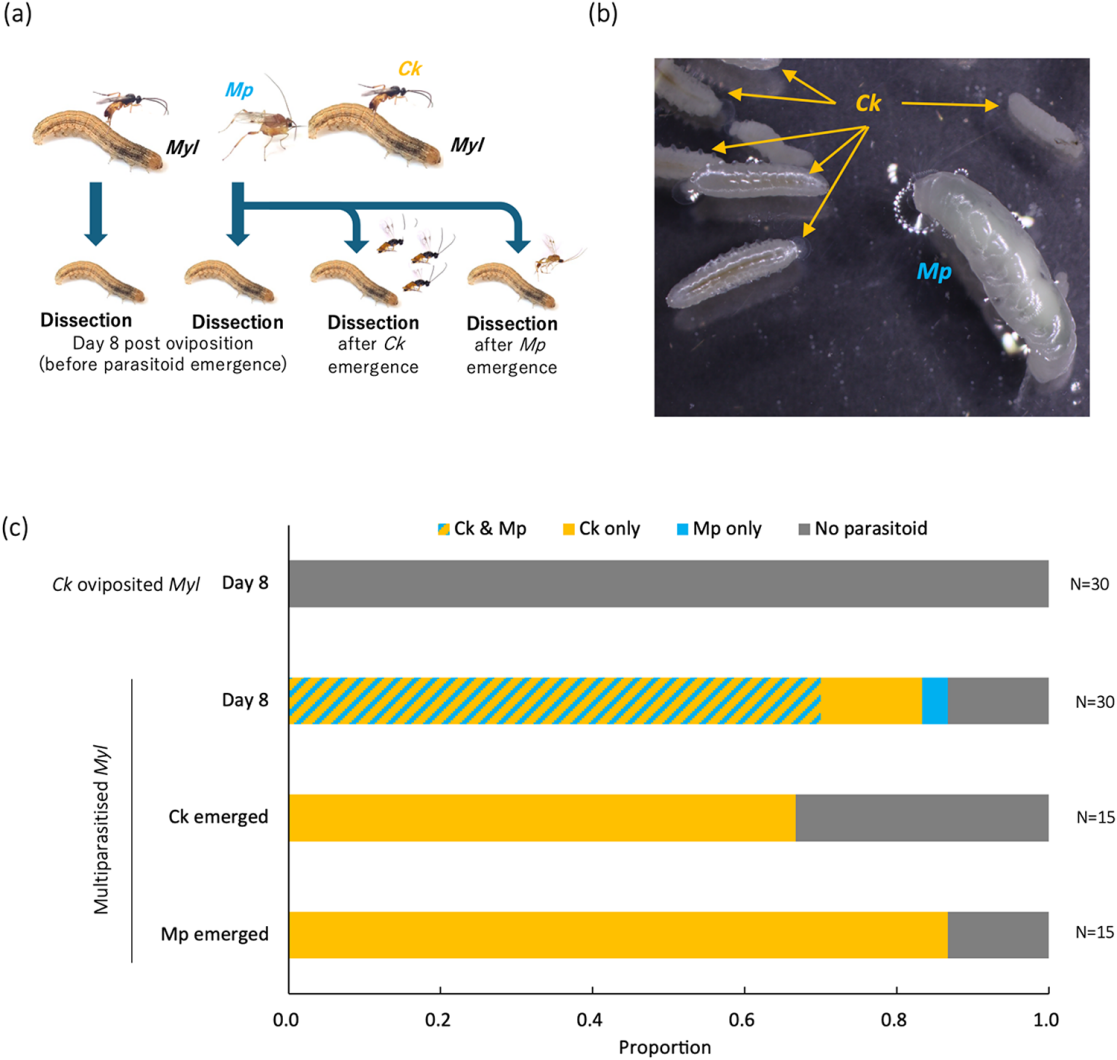
Observation of parasitoid larvae in *Mythmna loreyi* caterpillars. Preparation and dissection timing of parasitized caterpillars used for observation (**a**) and observed larvae of two parasitoids in multiparasitized *Myl* caterpillar (**b**). Proportion of observed parasitoid larvae is shown in (**c**). *Myl* = *Mythimna loreyi*, *Ck* = *Cotesia kariyai*, and *Mp* = *Meteorus pulchricornis.*

*Myl* caterpillars were dissected in four conditionsFig. 3a) and parasitoid larvae were observedFig. 3b). No *Ck* larvae were observed in *Ck*-oviposited *Myl* caterpillars 8 days post-ovipositionFig. 3c). In the case of day 8-multiparasitized *Myl* caterpillars, living *Ck* larvae were observed in 83.3% of caterpillars. Among these, 70.0% of caterpillars contained both living *Ck* and living *Mp* larvae whereas 13.3% contained only *Ck* larvaeFig. 3c). No *Ck* larvae were observed in 16.7% of the caterpillars, whereas 3.3% contained only *Mp* larva and the remaining 13.3% contained no parasitoid larvaeFig. 3c). In dead multiparasitized *Myl* caterpillars from which *Ck* emerged, dead *Ck* larvae were observed in 66.7% of the caterpillars, whereas the remaining caterpillars contained no *Ck* larvae. No *Mp* larvae were observed in these cases. In dead multiparasitized *Myl* caterpillars from which *Mp* emerged, dead *Ck* larvae were observed in 86.7%, while the others contained no parasitoid larvae. Hyperparasitism, in which *Ck* larvae parasitize in *Mp* larvae, was not observed (Fig. 3b).

### Experiment 6: simultaneous free-oviposition rearing in cages

**Fig. 4:**
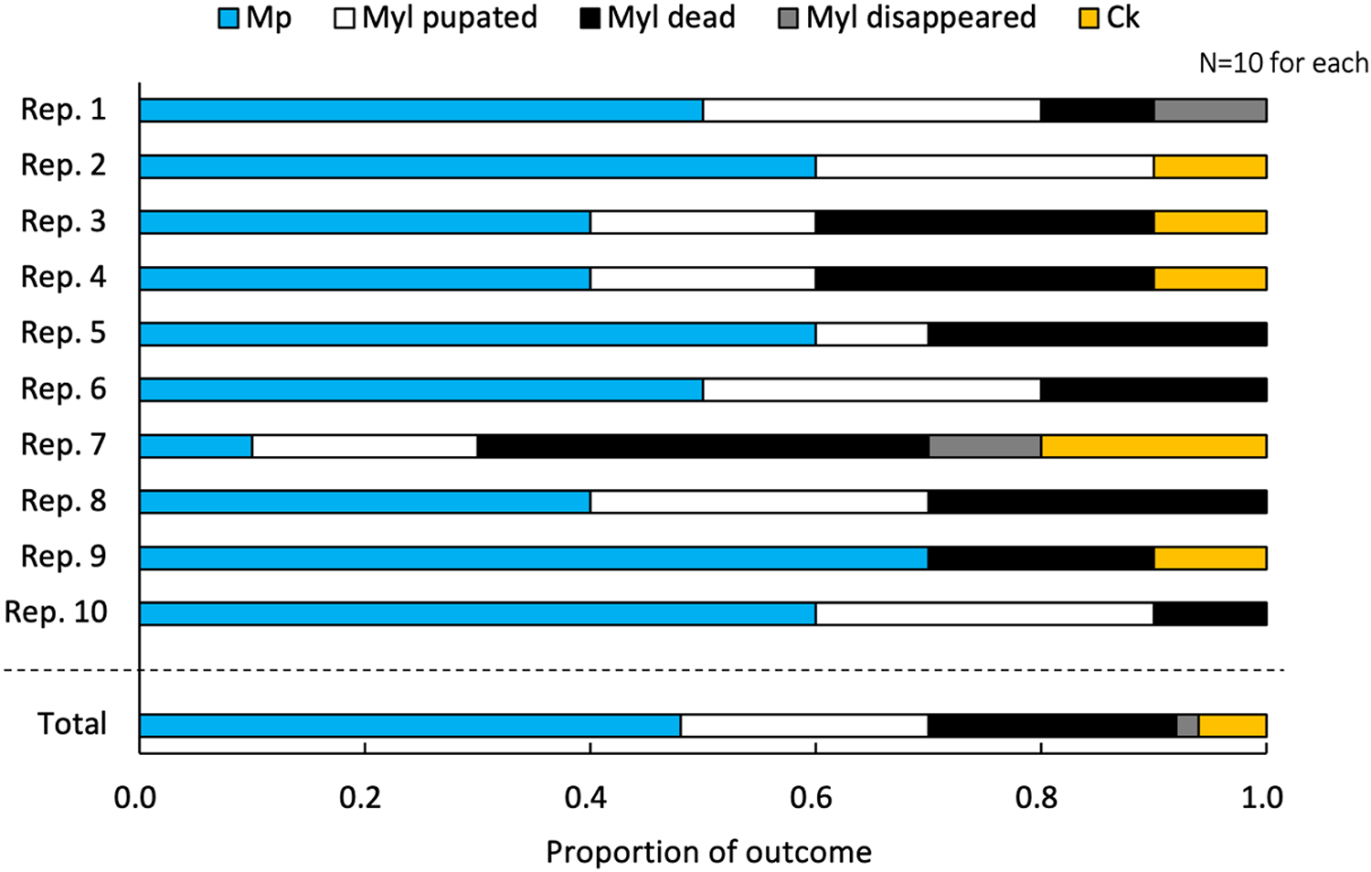
Outcome of simultaneous free-oviposition rearing experiments in cages. Results of each 10 replications and their total are shown.

Of 10 replicates, 5 showed successful parasitism of *Ck* in *Myl* caterpillars (Fig. 4). Overall, *Ck* emerged from 6.0% of *Myl* caterpillars. The number of *Ck* adults emerging from *Myl* caterpillars was 21.6 ± 9.3 (mean ± SD). *Mp* emerged from 48.0% of *Myl*. 22.0% of them pupate and another 22.0% died during experiments. Additionally, 2.0% of *Myl* caterpillars disappeared during cage experiments.

## Discussion

### Successful parasitism by *C. kariyai* in non-host *My*. *loreyi* parasitized by *Me.pulchricornis*

Attraction of parasitoid wasps to sympatric unsuitable host species and subsequent oviposition onto or into unsuitable hosts is widely documented. Females of the specialist endoparasitoid wasp, *C*. *kariyai* (*Ck*), also respond to chemical cues from unsuitable host species, including sympatric *M*y. *loreyi* (*Myl*), and exhibit oviposition behavior toward them^24,28,34,46^. Our results demonstrate that *Ck* females recognize *Myl* caterpillars and oviposit into them as into their usual host, *My. separata* (*Mys*) caterpillars (Experiment 1). However, *Myl* caterpillars parasitized solely by *Ck* never produced *Ck* adults (Experiment 2).

Remarkably, our multiparasitism experiments revealed that *Ck* can complete larval development in *Myl* caterpillars when these caterpillars are also parasitized by the naturally sympatric endoparasitoid wasp, *Me. pulchricornis* (*Mp*), which uses *Myl* as its usual host (Experiments 3 and 4). In essence, *Ck* can utilize unsuitable host *Myl* caterpillars as hosts when multiparasitism with *Mp* occurs. According to observations of parasitoid larvae in multiparasitized *Myl* caterpillars, no *Ck* larvae were found parasitizing *Mp* larva (Experiment 5). Therefore, hyperparasitism (cf.^65^), does not explain this phenomenon.

*Ck* females protect their eggs and their larvae from the immune response of their usual hosts, *Mys* caterpillars, by injecting polydnavirus (CkPDV) and venom^46,50,55,66^. These maternal anti-immunity factors are effective against *Mys* caterpillars, but not against other caterpillars^46^, which restricts the range of potential hosts for *Ck*. In fact, although *Ck* females oviposited 53.7–59.0 eggs into *Myl* caterpillars (Experiment 1), no *Ck* larvae were observed in hemocoels of *Myl* caterpillars eight days post-oviposition (Experiment 5), and successful parasitism never occurred (Experiment 2). Similarly, *Mp* females introduce virus-like particles (MpVLPs) into host caterpillars along with a single egg, which then regulate the immune response of the host insects^51–53^. Since 83.3% of multiparasitized *Myl* caterpillars contained *Ck* larvae eight days after oviposition, it is possible that maternal factors of *Mp* protect not only *Mp* eggs and larvae, but also *Ck* eggs and larvae, allowing *Ck* larvae to survive in *Myl* caterpillars. However, identifying physiological mechanisms that allow *Ck* to survive in unsuitable host caterpillars in multiparasitism remains a future challenge.

A series of studies by Magdaraog’s group^56–58^ clarified intrinsic competition between *Ck* and *Mp* in *Mys* caterpillars, which are ordinary hosts for both parasitoids. They demonstrated that multiparasitized *Mys* produce either *Ck* or *Mp* when the interval between the first and second oviposition is between 1 and 96 h, but the parasitoid species ovipositing first generally prevails over the other^56^. For example, when *Ck* oviposited into *Mys* 1 h before *Mp*, the successful parasitism rate of *Ck* was approximately 35%, which is significantly higher than that of *Mp*^56^. In contrast, in our study, the successful parasitism rate of *Ck* in multiparasitized *Myl* is significantly lower than that of *Mp*, regardless of oviposition order or host instar, with the maximum successful parasitism rate of *Ck* being only 20.0%, when *Ck* oviposited into 4th instar *Myl* caterpillars 12 h before *Mp* (Experiment 4).

The outcome of intrinsic competition between *Ck* and *Mp* in *Mys* was also mutually exclusive^56^, as observed in *Myl* in this study, and is determined by three factors: resistance to the host immune response, direct conflict among larvae, and toxic effects of maternal anti-immunity factors (CkPDV, MpVLP or venom) on heterospecific parasitoid larvae^58^. Our results showed that multiparasitized-*Myl* have the ability to produce *Ck* when female *Ck* oviposited *Myl* caterpillars < 24 h before *Mp*, or < 12 h after *Mp*. In cases in which *Ck* oviposited more than 48 h before *Mp*, *Ck* eggs may have been killed by the *Myl* immune response before *Mp* oviposition. Conversely, when *Ck* oviposited more than 48 h after *Mp*, *Ck* could not complete larval development because *Myl* caterpillars die soon after *Mp* emergence^67^. This assumption is supported by observation of dead *Ck* larvae inside *Myl* caterpillars from which *Mp* had emerged^67^.

Kraaijeveld^31^ hypothesized that oviposition into sympatric, unsuitable host species by parasitoids could increase survival probability when the ordinary host species is absent, acting as a kleptoparasitoid by exploiting the anti-host immunity of another parasitoid species via multiparasitism. However, successful parasitism of a parasitoid in unsuitable host species when multipatrasitised with a naturally sympatric parasitoid had not been demonstrated previously^31,32,42–44^. Our results present successful parasitism by a wasp in an unsuitable host through multiparasitism with a naturally sympatric parasitoid. Although the parasitism success rate by *Ck* in *Myl* caterpillars multiparasitized with *Mp* (< 20.0% ) was lower than that in the ordinary host *Mys* (> 70%^24,68^), oviposition by *Ck* females in unsuitable host *Myl* caterpillars is clearly possible. Therefore, as Kraaijeveld^31^ predicted, oviposition of parasitoid wasps in unsuitable hosts can be adaptive, especially when females cannot find their ordinary hosts. For example, in the case of *Ck* in Japan, the ordinary host, *Mys*, cannot overwinter when the average winter temperature falls below 4 °C, so the *Mys* population in spring is typically low. However, their numbers increase during summer and autumn due to mass migrations from China (Koyama & Matsumura^69^ and its references). Under these conditions, temporary use of “non-host” species like *Myl*, which occur stably throughout the year, may enhance the reproductive potential of *Ck*. Given that the parasitism rate of *Mp* on *Myl* in the wild can reach 42.9%^47^, it is likely that multiparasitism with *Ck* occurs. However, the emergence rate of *Ck* from *Myl* in cage experiments (Experiment 6) was extremely low (6%), and currently, there are no records of *Ck* emerging from *Myl* in the wild. The frequency of this phenomenon occurring in natural environments is likely extremely low, and further field investigations are required to document this interaction.

### Pirate parasitism: introducing a new term

Over 100 years ago, Pemberton and Willard^43^ discovered that the larval parasitoid wasp *Tetrastichus giffardianus* (Hymenoptera: Eulophidae), introduced into Hawaii from West Africa^70^, could use a local, unsuitable host, the Hawaiian fruit fly, *Bactrocera cucurbitae* (= *Dacus cucurbitae*) (Diptera: Tephritidae), only when the fruit fly larva was previously parasitized by another parasitoid, *Psyttalia fletcheri* (= *Opius fletcheri*) (Hymenoptera: Braconidae), also introduced to Hawaii from India^71^. Based on this study, Askew^72^ called this phenomenon “obligatory multiparasitism”, without providing a definition. On the other hand, today, the term “obligatory multiparasitism” (or obligate multiparasitism) is also used to describe a phenomenon observed in species such as *Pseudorhyssa* (Hymenoptera: Ichneumonidae) or *Eurytoma* (Hymenoptera: Eurytomidae)^73,74^. These wasps parasitize insects hidden in hard materials, such as timber or thick cocoons, as their hosts. However, they cannot drill through these materials themselves to reach the hosts. Therefore, these parasitoids attack hosts previously oviposited by another parasitoid by following an oviposition hole in the material created by the previous parasitoid. This behavior is often termed “obligate multiparasitism”^73,74^. Consequently, the term “obligatory parasitism” currently connotes parasitism that cannot be completed without the help of oviposition by another parasitoid.

**Fig. 5:**
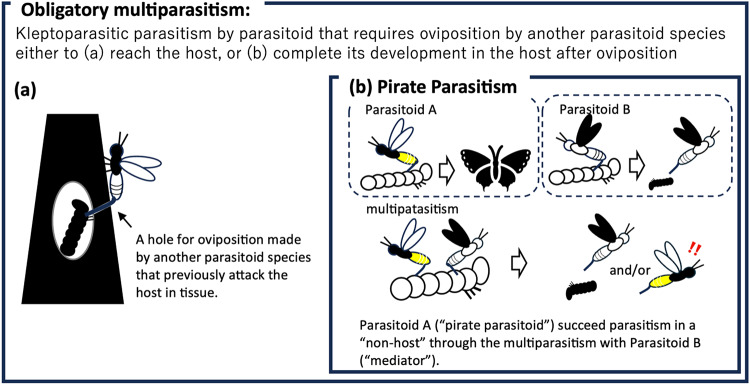
Pirate parasitism: Introducing a new term. Obligatory multiparasitism refers to kleptoparasitic parasitism by a parasitoid that requires oviposition by another parasitoid species either to (**a**) reach the host, or (**b**) complete its development in the host after oviposition. In the latter case, termed “pirate multiparasitism”, parasitism is defined as occurring when a parasitoid necessitates prior or subsequent parasitism by another parasitoid species and can complete its development through multiparasitism.

To avoid confusion, we propose a redefinition of the term “obligatory parasitism” and introduce a new term, “pirate parasitism” (Fig. 5). We redefine “obligatory multiparasitism” as kleptoparasitic parasitism by a parasitoid that requires oviposition by another parasitoid species to attack the host and/or complete development in/on the host body post-oviposition. In the latter case, as a subclass of obligatory multiparasitism, we propose a new term, “pirate parasitism”, defined as parasitism by parasitoids that necessitates prior parasitism by another parasitoid species, resulting in complete development through multiparasitism. We also propose referring to parasitoids that require multiparasitism to utilize an “unsuitable host” as “pirate parasitoids.” We call those parasitoid wasps that facilitate pirate parasitoids as “mediators” (Fig. 5).

In the case of the host/parasitoid system in our study, *C*. *kariyai* is a specialist parasitoid on *My*. *separata*, but can utilize *My*. *loreyi* through “pirate parasitism” when multiparasitism with *Me*. *pulchricornis* occurs. In this case, *C*. *kariyai* is the “pirate parasitoid”, and *Me*. *pulchricornis* is the “mediator.” *Meteorus pulchricornis*, suffers a disadvantage due to pirate parasitism. However, there may also be cases of pirate parasitism in which the mediator suffers no disadvantage.

### The ecological and evolutionary significance of the response and oviposition in unsuitable host species

The concept of pirate parasitism indicates that attraction to, acceptance of, and oviposition by parasitoids on unsuitable host species increases reproductive potential. If parasitoid wasps were only attracted to their customary hosts, their reproductive success would be reduced to zero whenever their usual host was absent. However, with pirate parasitism, parasitoids can oviposit in unsuitable hosts, thereby maintaining some reproductive potential and increasing their fitness. Specialist parasitoid wasps, in particular, are more likely than generalist parasitoids to encounter environments in which their hosts are absent. Therefore, reproducing via pirate parasitism might significantly increase their reproductive potential. This suggests that the seemingly maladaptive behavior of parasitoid wasps ovipositing in non-hosts may actually represent an adaptive behavior. In fact, many species of parasitoids are attracted to and oviposit not only in sympatric unsuitable hosts, but also in introduced unsuitable hosts (Kruitwagen et al.,^75^ and references).

The concept of pirate parasitism is also important from the perspective of evolution of parasitoid host ranges. Host shifts and host range expansions of parasitoid wasps have been thought to occur through a process in which wasps accidentally oviposit in unsuitable host that they had not previously used^2,37–39^. If development is successful in unsuitable hosts, newly emerged wasps learn the smell of that new host or its habitat, and subsequently show a preference for oviposition on it. The fact that our research results indicate that oviposition in unsuitable hosts can increase reproductive success implies that such oviposition behavior can serve as preadaptation in the context of host shifts and host range expansions in parasitoid wasps. This could increase the likelihood of the aforementioned “happy accident.”

Multiparasitism is also generally considered maladaptive for parasitoids as it reduces the likelihood of successful parasitism due to direct or indirect competition between parasitoid species^76^. However, in the case of pirate parasitism, a pirate parasitoid increases its fitness through multiparasitism. This suggests that female pirate parasitoids can identify and preferentially oviposit in unsuitable hosts that have already been parasitized by mediators, but this possibility remains to be verified.

Even though multiparasitism is thought to occur frequently in the field, it is difficult to detect by host collection and rearing because in most cases, only one parasitoid species emerges from the host^76^. Future studies need to investigate the presence, frequency, and generality of pirate parasitism in parasitoid-host systems by detecting multiparasitism using molecular tools with species-specific primers, as recently used for detection of hyperparasitism^77,78^ and laboratory multiparasitising experiments.

## Materials and methods

### Insects

*Mythimna separata* and *C*. *kariyai* were obtained from stock cultures in the Laboratory of Applied Entomology and Zoology, University of Tsukuba, Japan. Caterpillars of *Mythimna loreyi* were collected from maize and sorghum fields in Kimotsuki-cho, Kanoya City and Kagoshima City, Kagoshima Prefecture, Kyushu, Japan, between July 28 and 30, 2020. The original colony of *Meteorus pulchricornis* was obtained from *M. separata* caterpillars collected in the field, as described above. Both *Mys* and *Myl* colonies were reared on an artificial diet (Silkmate^®^ 2 S, Nosan Corporation, Yokohama, Japan) in the laboratory (25 ± 1 °C, 16 L: 8D photoperiod and 60 ± 10% RH) following methods for *Mys* described in Fukushima et al.^79^ and Magdaraog et al.^56^ under the same conditions as the herbivores. Populations of both parasitoid species have been maintained using 3rd instar *Mys* caterpillars as hosts under laboratory conditions. For *Ck*, three to five-day-old mated, naïve females were used for all experiments. Because the *Mp* strain we used was an apomictic thelytokous strain (cf. Fujie et al.,^80^; Wachi et al.,^81^), unmated, 7-10-day-old females were used for all experiments.

### Experiment 1: host acceptance and oviposition

Female parasitoids with no oviposition experience were used for the experiment. Each female parasitoid was placed individually in a Petri dish (5.3 cm diameter, 1.5 cm height) containing wet cotton 30–60 min prior to the experiment. A single caterpillar was then introduced into the Petri dish, and parasitoid’s behavior was observed for 10 min. Whether the parasitoid attacked the caterpillar was recorded. Throughout experiments in this study, oviposition was confirmed by observing a single insertion and withdrawal of the ovipositor from the caterpillar. For *Ck*, 3rd, 4th or 5th instar unparasitized *Myl* caterpillars or *Myl* caterpillars previously oviposited by *Mp* were offered. Similarly, 3rd, 4th or 5th instar unparasitized *Myl* caterpillars or *Myl* caterpillars previously oviposited by *Ck* were offered to *Mp*. Parasitized *Myl* caterpillars used for these experiments were prepared by single oviposition by parasitoids ca. 1 h before experiments. As a positive control for *Ck* oviposition, 4th instar, unparasitized *Mys* caterpillars (ordinary host for *Ck*) were also offered.

To confirm the presence of parasitoid egg(s) in the caterpillar body, caterpillars were dissected within 30 min after oviposition using the method of Aikawa et al.^34^. Numbers of parasitoid eggs in the caterpillar were recorded. Dissected caterpillars were observed under a binocular stereo microscope (SMZ 1270, Nikon, Tokyo, Japan).

### Experiment 2: single-parasitism experiments

Host suitability of *Myl* caterpillars for the two parasitoids when each parasitoid oviposited alone was determined. Parasitoids with no prior oviposition experience were allowed to oviposit into 3rd, 4th or 5th instar *Myl* caterpillar in a Petri dish. The oviposited caterpillar was then transferred into a plastic container (100 mm in diameter, 40 cm in height, Sansho, Tokyo, Japan) and reared individually with artificial diet until the parasitoid emerged, the caterpillar pupated, or the caterpillar died.

### Experiment 3: multiparasitism experiments in different host instars

To examine successful parasitism of *Ck* in *Myl* caterpillars parasitized with *Mp*, the outcome of multiparasitism with both parasitoids in 3rd, 4th or 5th instar *Myl* caterpillars was observed. Multiparasitized *Myl* caterpillars, with oviposition by *Ck* and *Mp* in either order in < 15 min, were prepared using methods described for Experiment (1). Parasitized caterpillars were reared individually, and outcomes were recorded as described for Experiment (2). When *Ck* emerged from *Myl* caterpillars, to verify their reproductive ability, five newly emerged female adults from each treatment were allowed to oviposit into their usual host, *Mys*, after mating. Parasitized *Mys* caterpillars were reared individually as described above, and outcomes were recorded using the methods described in the single-parasitism experiment section.

For positive control for *Ck* parasitism, 4th instar *Mys* was also offered to *Ck* and oviposited *Mys* caterpillars were reared individually as same as *Myl c*aterpillars. Then, the number of parasitoids per caterpillar was counted.

### Experiment 4: multiparasitism in different sequences and intervals

To examine effects of the order and timing of oviposition by the two parasitoid species on outcomes of multiparasitism in *Myl* caterpillars, 4th instar caterpillars parasitized by the two parasitoid species in different sequences and intervals were reared. Both parasitoids were allowed to oviposit into caterpillars in either order of oviposition, at intervals of < 10 min, 12 h, 24 h, and 48 h. Parasitized caterpillars were reared individually, and outcomes were recorded using methods described above.

### Experiment 5: observation of parasitoid larvae in multiparasitized caterpillars

Based on results of multiparasitism experiments, multiparasitized *Myl* caterpillars produced not only *Mp*, but also *Ck*. To ascertain whether hyperparasitism occurred involving the two parasitoid species in the same *Myl* caterpillars, multiparasitized *Myl* caterpillars were dissected and parasitoid larvae in the caterpillars were observed. Multiparasitized 4th instar *Myl* caterpillars, which were oviposited by *Ck* followed by *Mp* within < 10 min, were prepared using methods described previously. Parasitized caterpillars were reared on artificial diet. Caterpillars were dissected in 70% ethanol-water solution before parasitoid emergence (on day 8 after oviposition) or within 12 h after parasitoid emergence to observe the presence or absence of parasitoid larvae, and whether hyperparasitism occurred between the two parasitoid species. Additionally, *Myl* caterpillars oviposited by *Ck* alone were also dissected on day 8 after oviposition to determine whether *Ck* larvae survived in *Myl*. All observations were conducted under a binocular stereo microscope (SMZ 1270, Nikon, Tokyo, Japan).

### Experiment 6: simultaneous free-oviposition rearing in cages

To examine whether *Ck* could emerge from *Myl* in an environment in which it could freely oviposit, we created a rearing cage in which *Ck*, *Mp*, and *Myl* coexisted and evaluated the emergence rate of *Ck* from *Myl*. This experiment was conducted in transparent plastic containers (17 × 19 cm, 29 cm high). Maize (“Honey-Bantam Peter 610”, Sakata Seed Co., Japan) was utilized as the host plant for *Myl* caterpillars. Maize plants were cultivated from seed in plastic flowerpots (14.0 cm diameter, 11.5 cm height) in a greenhouse (25 ± 1 °C, 16 L:8D photoperiod)^82^. Stems of 3- to 4-week-old maize plants containing 3–4 leaves, approximately 25 cm high, were cut and placed in a 50-mL beaker filled with water. Maize leaves were placed at the center of the container, and ten 4th instar *Myl* caterpillars were placed on the leaves. Three females each of both *Ck* and *Mp* were released into the container. Honey and wet cotton were provided in the container for the wasps. These containers were maintained in a rearing room (25 ± 1 °C, 16 L: 8D photoperiod and 60 ± 10% RH) for 48 h. All caterpillars were then collected from the container and reared individually, and outcomes were recorded using the same methods described in the singleparasitism experiment section.

### Statistical analysis

For experiment 1, differences in host acceptance rates of parasitoids among treatments were evaluated with Chi-square tests, and differences in numbers of eggs oviposited in caterpillars among caterpillar conditions were evaluated using analysis of variance (ANOVA). For experiment 2, the successful parasitism rate among host instars was evaluated with Chi-square tests. In experiment 3, the significance of the emergence percentage of the two wasps in each host instar and order of oviposition was analyzed using binominal tests. The successful parasitism rate of *Ck* among host instars and the order of oviposition were evaluated with Chi-square tests. Mean numbers of *Ck* adults that emerged from caterpillars were evaluated using Tukey-Kramer honestly significant difference (HSD) tests after ANOVA. In experiment 4, the successful parasitism rate of *Ck* by order and interval of oviposition was evaluated with Tukey’s wholly significant difference (WSD) tests after Chi-square tests, and mean numbers of *Ck* adults emerging from caterpillars were evaluated using Tukey-Kramer HSD tests after ANOVA. All analyses were performed in R v. 4.0.3 software^83^ and Tukey-Kramer HSD tests and Tukey WSD tests were performed using an open-source package (http://aoki2.si.gunma-u.ac.jp/R/m_multi_ comp.html). Since experiments 5 and 6 were qualitative experiments, statistical analyses were not conducted.

## Electronic supplementary material

Below is the link to the electronic supplementary material.

Supplementary Material 1

## Data Availability

The datasets generated during and/or analyzed during the current study are available from the corresponding author on reasonable request.
