# Supplementary material for: Multiparasitism enables a specialist endoparasitoid to complete parasitism in an unsuitable host caterpillar
Source: Sci Rep. 2025 Mar 11;15:8361. doi: 10.1038/s41598-025-91403-3 (PMC11897168; doi:10.1038/s41598-025-91403-3)
Supplement: Supplementary file 1 — Supplementary Material 1 [file 41598_2025_91403_MOESM1_ESM.pdf]

Supporting Information

Authors: Kazumu Kuramitsu, Yooichi Kainoh and Kotaro Konno

Title: Multiparasitism enables a specialist endoparasitoid to complete parasitism in an unsuitable host caterpillar

Figure S1

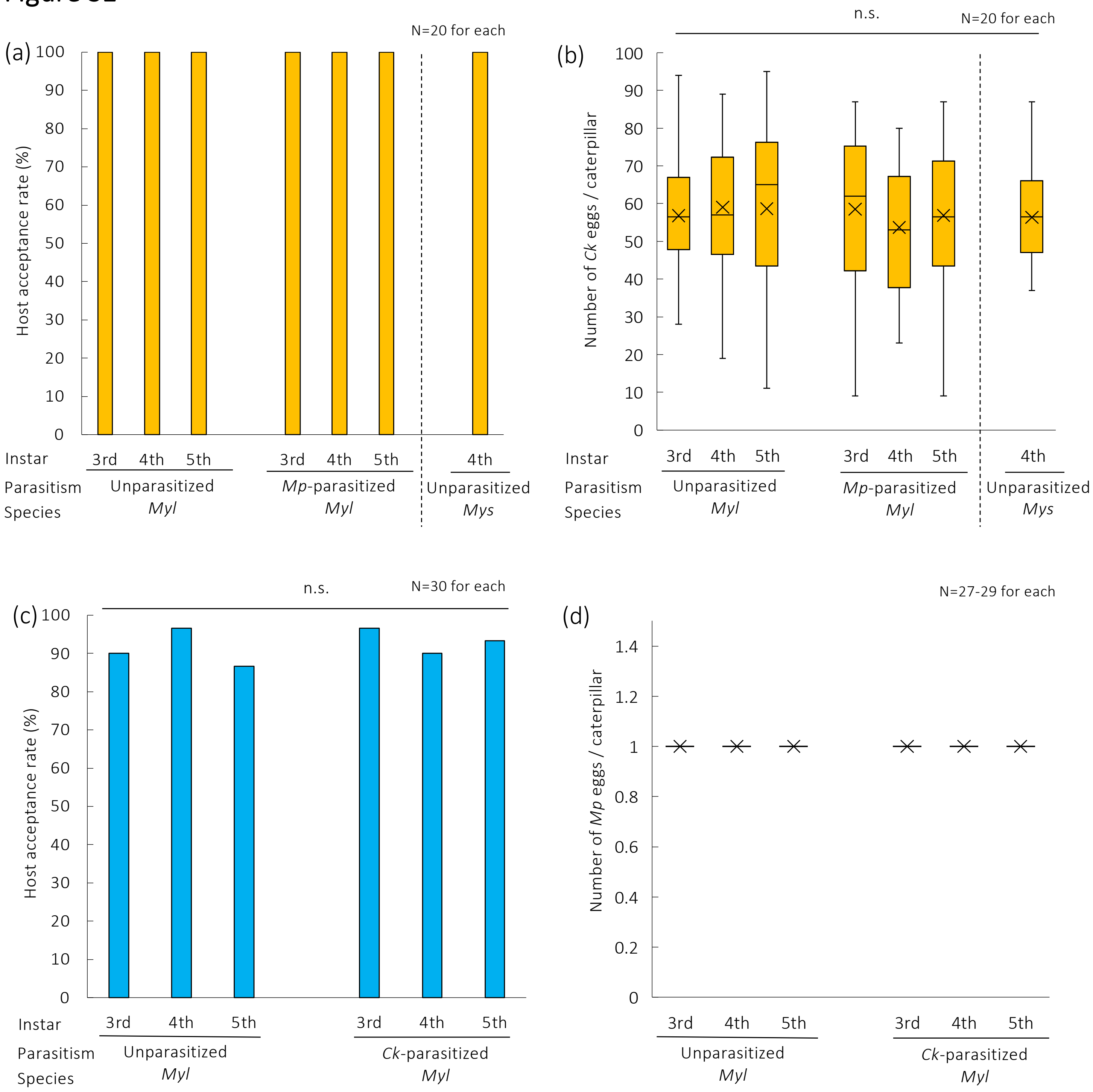

Supplementary Figure S1. Host acceptance and oviposition by *Cotesia kariyai* and *Meteorus pulchricornis* to unparasitized *Mythimna loreyi* caterpillars and caterpillars previously oviposited by another parasitoid.

Host acceptance (a, c) and number of eggs found in the caterpillar body (b, d) by *C. kariyai* (a, b) and *M. pulchricornis* (c, d). *Myl* = *Mythimna loreyi*, *Mys* = *My. separata*, *Ck* = *Cotesia kariyai*, and Mp = *Meteorus pulchricornis* . (n.s.: no significant differences, by ANOVA (b) or Chi-square test (c))

Figure S2

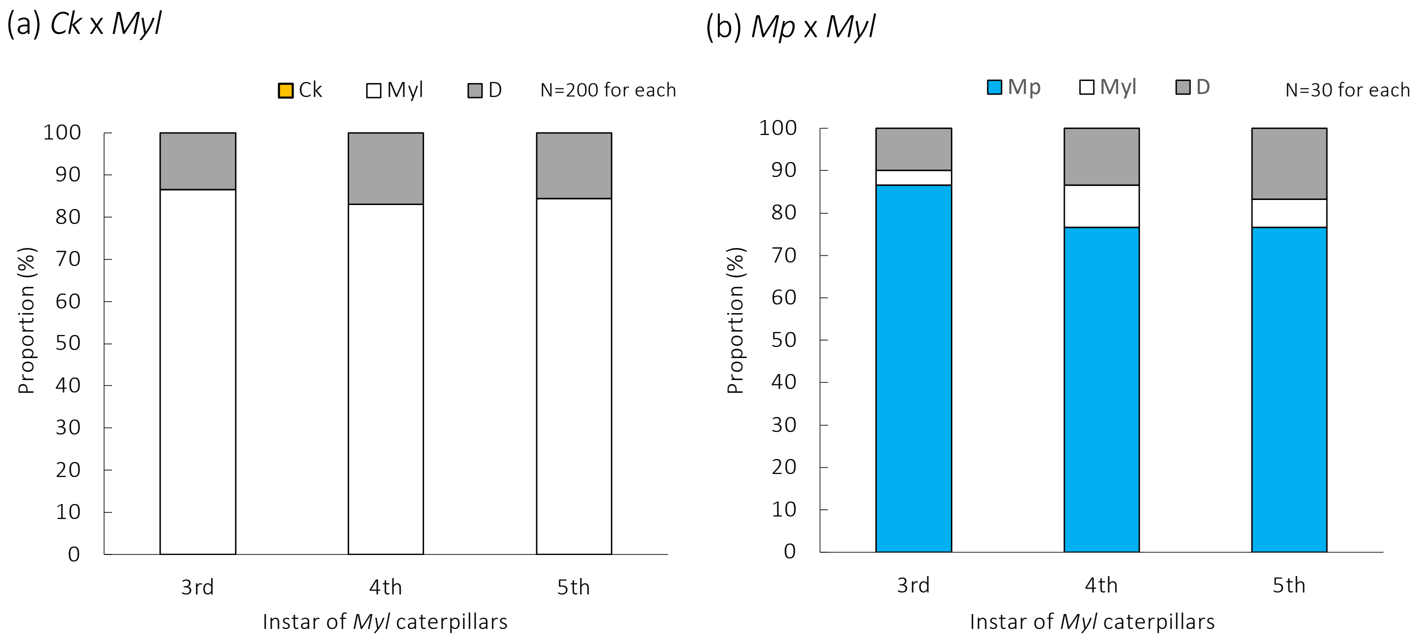

Supplementary Figure S2. Successful parasitism rate of *Cotesia kariyai* (a) and *Meteorus pulchricornis* (b) in *Mythimna loreyi* caterpillars. Female parasitoids were allowed to oviposit into 3rd, 4th or 5th instar *Myl* caterpillars, and oviposited caterpillars were reared individually to examine the host suitability.

Myl = *Mythimna loreyi*, *Ck* = *Cotesia kariyai* and *Mp* = *Meteorus pulchricornis.*

(n.s.: no significant differences by Chi-square test)

Figure S3

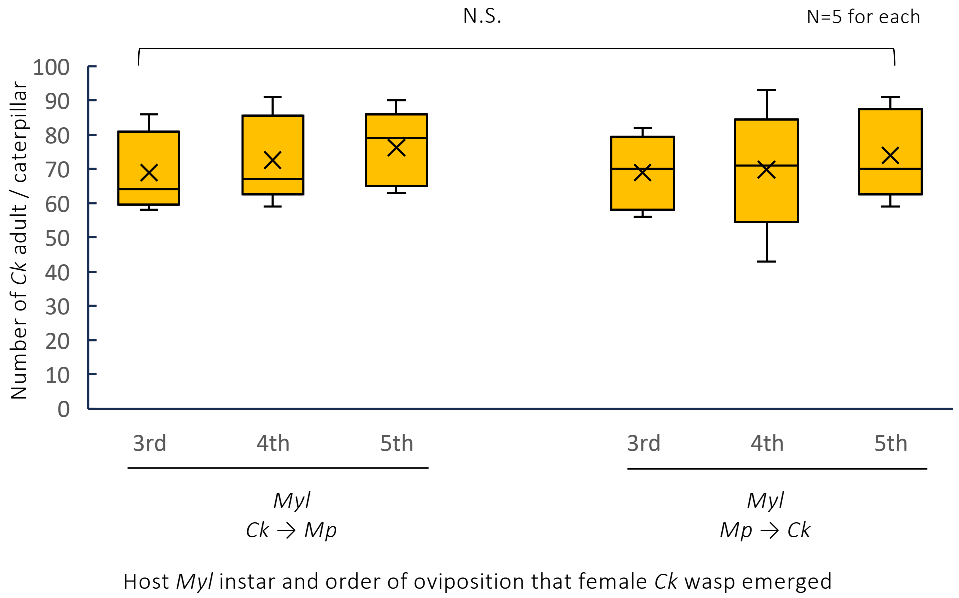

Supplementary Figure S3. Reproductive ability of *Cotesia kariyai* emerged from *Mythimna loreyi*. Number of emerged *Ck* wasps per *Mythimna separata* caterpillar after oviposition by *Ck,* which emerged from *Myl* under different multiparasitism conditions (instar and order of oviposition by two parasitoids).

Myl = *Mythimna loreyi*, *Mys* = *My*. *separata, Ck* = *Cotesia kariyai* and *Mp* = *Meteorus pulchricornis.* (n.s.: no significant differences by ANOVA)
